# Filling the gaps: Original chronologies of silver fir (*Abies alba* Mill.) and European beech (*Fagus sylvatica* L.) living trees in the French Pyrenees

**DOI:** 10.1016/j.dib.2025.111596

**Published:** 2025-05-03

**Authors:** Mélanie Saulnier, Vincent Labbas, Roberta D’Andréa, Sylvain Burri, Laurent Larrieu, Sylvain Morvan, Nicolas Martin, Caroline Scotti-Saintagne, Frédéric Jean, Nicolas Poirier, Vanessa Py-Saragaglia

**Affiliations:** a CNRS UMR 5602 GEODE, Université Jean Jaurès, 5 allées Antonio Machado, Toulouse Cedex 9, 31058, France; b University of Liege, Place du Vingt Août 7, Liège 4000, Belgium; c Royal Institute of Art Heritage, Parc du Cinquantenaire 1, Brussels 1000, Belgium; d CNRS, UMR 5608 TRACES, Université Jean Jaurès, 5 Allée Antonio Machado, Toulouse Cedex 9, 31058, France; e UMR 1201 DYNAFOR, University of Toulouse, INRAE, INPT, EI Purpan, 24 Chemin de Borde Rouge, Auzeville‑Tolosane 31320, France; f INRAE, UMR Écologie des forêts méditerranéennes, URFM, Site Agroparc, Domaine Saint-Paul, Avignon Cedex 9, 84914, France; g UMR 7065 IRAMAT-LMC, Université de technologie de Belfort-Montbéliard, Campus de Sévenans, Belfort Cedex, 90010, France

**Keywords:** Dendrochronology, Reference chronologies, French Pyrenees, *Abies alba* Mill*.*, *Fagus sylvatica* L*.*, Old-growth forests, Ancient forests, Managed forests

## Abstract

Between 2016 and 2024, as part of several research projects on forest history in the mountain belt of the central French Pyrenees, we cored 360 living trees at breast height, including 309 silver fir (Abies alba Mill. 1768) and 51 European beech (Fagus sylvatica L. 1753), using an increment drill. In addition, 64 cross-sections of beech stems were harvested with a chainsaw during a forest cut by the forest manager. The trees are located in 14 stands (13 fir-dominated forests with some beech, 1 beech-dominated forest, in 3 stands we sampled both species) spread over the French Central Pyrenees and subject to different levels of harvesting pressure: regularly harvested, harvested 30-40 years ago, not harvested for a long time (>100 years). In each stand, we established three to five circular plots of 1 ha each. Depending on the main objective of the research projects, trees were selected from the largest, dominant or co-dominant individuals according to a pre-established protocol, as for the BENDYS project [1] (full description in Py-Saragaglia et al., 2020), or to represent the full range of diameter at breast height (DBH) available in the 1 ha circular plots. All samples were processed using standard dendrochronological techniques at the GEODE laboratory in Toulouse (PANGEME analysis platform). Tree-ring measurements were made with a resolution of 0.001 mm using either a LINTAB sliding table connected to TsapWin software (RINNTECH, Heidelberg, Germany) or CooRecorder [2] software applied to a high-resolution scan of the samples. All samples were cross-dated using CDendro [2] software. The robustness of the chronologies was assessed using reference curves available for both species in the Pyrenees for beech and in southern France for fir.

The present dataset provides two reference chronologies for fir and beech in this part of the Pyrenees and 17 master stand chronologies, 13 from fir and 4 from beech. The reference chronologies were constructed from 324 of the 424 individual ring series sampled, 253 from fir (309 sampled) and 71 from beech (115 sampled). All reference chronologies are suitable for dendroarchaeological studies (dating, dendroprovenance, reconstruction of ancient practices, etc.), dendroecological studies (resilience of species, effect of management on growth, etc.) and dendroclimatological studies (climate reconstruction, climate sensitivity of the two species, etc.).

Specifications Table

**Table:** 

Subject	*Ecology*
Specific subject area	Original reference and master stand tree-ring chronologies from silver fir and European beech living trees acquired in the French central-eastern Pyrenees
Type of data	Tables (.csv format) Heidelberg Format Ring Width files (.fh or .rwl format) R.file (.R) Supporting materials: “Codebook” to use data (.txt format) and 2 figures
Data collection	This article presents original reference and local master stand tree-ring chronologies from fir and beech living trees acquired since 2016 in 14 stands located in the French central Pyrenees. The samples were collected either as tree cores (using an increment borer) or cross-sections (using a chainsaw). Ring widths were measured at a resolution of 0,001 mm using a LINTAB sliding table connected to TsapWin software (RINNTECH, Heidelberg, Germany), or using CooRecorder software applied to a high-resolution scan of the samples. Cross-dating was conducted using CDendro software programs, and the chronologies exhibiting the weakest correlations were excluded from the analysis. We subsequently built the master stand chronologies and reference chronologies for the Pyrenees. All master stand and reference chronologies are suitable for dendroarchaeological, dendroecological and dendroclimatological studies.
Data source location	Central and eastern part of the French Pyrenees Data are stored in the GEODE Laboratory (CNRS UMR 5602 Toulouse, France)
Data accessibility	Repository name: https://data.indores.fr/ Data identification number: 10.48579/PRO/JQQRRK Direct URL to data: https://doi.org/10.48579/PRO/JQQRRK Instructions for accessing these data: open access
Related research article	Fouédjeu L., **Saulnier M.**, Lejay M., Dušátko M., Labbas V., Jump A. S., Burri S., Buscaino S., Py-Saragaglia V., 2021. High resolution reconstruction of modern charcoal production kilns: An integrated approach combining dendrochronology, micromorphology and anthracology in the French Pyrenees. Quaternary International, 593: 306-319*.*

## 1 Value of the Data

• The data provide unique fir and beech tree-ring chronologies at large spatial scale in the French Pyrenees built using 424 living trees.
• The availability and sharing of dendrochronological dataset are essential for refining the research carried out using these proxies, such as climate reconstructions.
• The data can be reused for any dendrochronological purposes, i.e. dendroclimatology, dendroarchaeology (dating, dendroprovenancing, building history, land use etc.) and dendroecology
• The master chronologies constructed on stands subjected to different degrees of harvesting pressure (regularly harvested, harvested 30-40 years ago, long time (>100 y) unharvested) could also be employed to evaluate the impact of human activity on the resilience of the fir to climatic conditions in the Pyrenees. This would facilitate the formulation of management policies, either aimed at maintaining a high forest silvicultural approach or at transitioning towards more natural management.

## 2 Background

The French Pyrenees, like much of southwestern France, is characterized by a profound lack of dendrochronological references [3]. In recent years, several projects have focused on forest ecosystems history and dynamics in the Pyrenees, with funding (from the ANR and the Occitanie Region) allocated to dendrochronological sampling campaigns on living trees. Most of these projects, mainly led by CNRS researchers from the GEODE and TRACES Laboratories (Toulouse) and also INRAE researchers from URFM laboratory (Avignon), have begun to fill this reference gap by sampling fir and beech trees in forests characterized by different degrees of human pressure, from managed forest (Canigou, Burat, Bernadouze) to ancient forests currently managed as high stand forests (Aragnouet, Bois du Far, Burat, Canigou, Lercoul) and, ancient mature forests encompassing many old-growth forests attributes (Burat, Bois-Neuf, Barrada, Montious) since harvesting has ceased several decades ago. One dataset (Bernadouze) was acquired as part of Léonel Fouédjeu's PhD thesis and already allowed the publication of an original paper [4]. Some of the new reference chronologies have been the subject of oral presentations during international conferences. The fir reference chronology from the Barrada forest also accurately cross-dates with the dendroarchaeological chronology built from buildings sampled in the Gavarnie-Gedre valley [5].

## 3 Data Description

The repertory associated with this data-in-brief paper available in DataInDores includes 4 folders (with sometimes subfolders) and one text files:

• (1) The folder “Individual series and chronologies” provides master stand and reference chronologies as well as the tree-ring width series used to build these chronologies.
  ○ The subfolder ‘chronologies’ contains the 4 files with all master stand and reference chronologies in Tucson format (.rwl) and in tabular format (.csv) to make them as accessible as possible. The file entitled “Master stand and reference chronologies_AA”, in .csv or.rwl format, contains the master stand and reference chronologies for fir (Master_AA.fh or .csv). The file entitled “Master stand and reference chronologies_FS”, in .csv or.rwl format, contains the master stand and reference chronologies for beech (Master_AA.fh or .csv).
  ○ The subfolder ``Individual series for stand and Pyrenees'' contains individual tree-ring series used to construct reference and stand chronologies, classified by stand. The folder is divided into two subfolders according to the data format. These subfolders are also divided into two subfolders according to the species considered. Irrespective of the data format, the 'Abies alba' subfolder contains 14 files, 13 for each stand and 1 for the Pyrenees as a whole. The 'Fagus sylvatica' subfolder contains 5 files, 4 for each stand and 1 for the Pyrenees reference.
• (2) A folder containing two tables is also provided (in .tab format). Both tables include a “Read_me” sheet in which the reader will find all explanations and details regarding all variables and all abbreviations.
  ○ Table 1 provides the geographical characteristics (latitude, longitude and altitude) of the chronology sites, the full name of the locality and the forest, the short name of the population, the degree of anthropogenic pressure (HF: high stand forest; AF: ancient forest, OF: old-growth forest), the project in which the data were acquired and the number of trees cored at each site (Table 1_master stand caracteristics.csv).

**Table 1:** Site description of master stand and reference chronologies. The type of forest refers to the time since the last harvesting: > 100 yrs old-growth forests (OF); >50 yrs ancient forests (AF); <50 yrs high standard forests (HF). Species.

Species	Locality	Forest	Project	Short name population	Type of forest	Name master chronology	Lat	Lng	Alt.	Nb. sampled trees
**Abies alba**	Aragnouet*	Aragnouet	BOSCA	ARA	AF	ARA_OF0_AA	42.81	0.19	1700	30
	Gèdre	Barrada	BENDYS	BAR	OF	BAR_OF0_AA	42.81	0.03	1625	22
	Bois du Far	Bois du Far	TRANSYLVE	BDF	AF	BDF_AF0_AA	42.76	1.45	1400	8
	Saint Mamet	Bois-neuf	BENDYS	BNF	OF	BNF_OF0_AA	42.76	0.64	1750	20
	Marignac	Burat	Occigen	BUR	AF	BUR_AF0_AA	42.88	0.68	1450	25
	Marignac	Burat	Occigen	BUR	HF	BUR_HF0_AA	42.88	0.65	1400	25
	Marignac	Burat	BENDYS /OcciGen	BUR	OF	BUR_OF0_AA	42.87	0.67	1550	52
	Estoher	Canigou	Occigen	CAN	AF	CAN_AF0_AA	42.50	2.41	1750	24
	Estoher	Canigou	Occigen	CAN	HF	CAN_HF0_AA	42.50	2.4	1550	25
	Estoher	Canigou	Occigen	CAN	OF	CAN_OF0_AA	42.56	2.47	1550	22
	Hourmigué	Hourmigué	BENDYS	HOU	OF	HOU_OF0_AA	42.92	0.6	1400	8
	Lercoul	Lercoul	TRANSYLVE	LER	AF	LER_AF0_AA	42.76	1.54	1500	23
	Bordères-Louron	Montious	BOSCA	MON	OF	MON_OF0_AA	42.87	0.47	1700	25
	***Total number of fir trees sampled in Pyrenees***									***309***
**Fagus sylvatica**	Gèdre*	Barrada	BENDYS	BAR	OF	GED_OF0_FS	42.81	0.03	1625	19
	Suc-et-Sentenac	Bernadouze	FODYNA	BER	HF	BER_HF0_FS	42.8	1.42	1500	64
	Saint Mamet	Bois-neuf	BENDYS	BNF	OF	BNF_OF0_FS	42.76	0.64	1750	15
	Marignac	Burat	BENDYS	BUR	OF	BUR_OF0_FS	42.87	0.67	1300	17
	***Total number of beech trees sampled in Pyrenees***									***115***
  ○ Table 2 provides the main statistics calculated on the raw chronologies, the number of individual series kept building master stands or reference chronologies, the mean and standard deviation (stdev) of ring-widths, and the first order autoregression (AR1). To calculate Rbar and EPS, individual series were detrended using an age-dependent spline curve to remove the age effect and an autoregression to remove the regressive effect (Table 2_Descriptive statistics.csv).

**Table 2:** Main statistics of master stand and reference chronologies.

Species	Stand short names	Chronologie names	Nb. Of individual series	Begin	End	Length	mean	stdev	Ar1	Rbar	EPS
**Abies alba**	ARA	ARA_AF0_AA	27	1740	2023	284	1.172	0.396	0.83	0.327	0.929
	BAR	BAR_OF0_AA	20	1784	2020	237	1.963	0.715	0.862	0.304	0.897
	BDF	BDF_AF0_AA	8	1764	2018	201	1.407	0.658	0.913	0.330	0.797
	BNF	BNF_OF0_AA	20	1780	2021	242	1.914	0.534	0.79	0.331	0.908
	BUR	BUR_OF0_AA	41	1835	2023	189	1.962	0.629	0.785	0.249	0.931
	BUR	BUR_AF0_AA	25	1881	2023	143	2.473	0.735	0.828	0.393	0.942
	BUR	BUR_HF0_AA	22	1831	2022	192	2.66	0.799	0.781	0.315	0.910
	CAN	CAN_AF0_AA	22	1846	2022	177	2.018	0.389	0.693	0.340	0.919
	CAN	CAN_OF0_AA	22	1903	2022	120	2.646	0.542	0.668	0.379	0.931
	CAN	CAN_HF0_AA	25	1864	2022	159	2.352	0.801	0.847	0.378	0.938
	HOU	HOU_OF0_AA	5	1851	2020	170	2.782	1.437	0.91	0.245	0.619
	LER	LER_AF0_AA	14	1827	2022	196	1.776	0.701	0.825	0.230	0.807
	MON	MON_OF0_AA	23	1605	2023	419	1.028	0.456	0.868	0.300	0.908
	***Pyr***	***Pyr_AA***	***253***	***1605***	***2023***	***419***	***1.439***	***0.711***	***0.930***	***0.204***	***0.985***
**Fagus sylvatica**	BAR	BAR_OF0_FS	15	1844	2020	177	1.136	0.405	0.623	0.243	0.828
	BER	BER_HF0_FS	46	1851	2016	166	1.539	0.633	0.777	0.394	0.968
	BNF	BNF_OF0_FS	8	1840	2020	181	1.054	0.416	0.43	0.278	0.755
	BUR	BUR_OF0_FS	5	1848	2021	174	1.392	0.862	0.861	0.215	0.578
	***Pyr***	***Pyr_FS***	***71***	***1840***	***2021***	***182***	***1.382***	***0.475***	***0.682***	***0.282***	***0.965***
• (3) The folder “Figures” includes four figures while we presented only two of them in this data paper (Figs. 1 and 2). The Fig. 1 is a map of the stand locations within the French Pyrenees, and the Fig. 2 provides a plot of detrended reference chronologies for both species. The Figs. 3 and 4 (only enclosed in the data repertory) represent the plot of all detrended master stand chronologies (Please pay close attention to the scale of the abscissa (years), which varies according to the chronology).

**Fig. 1:**
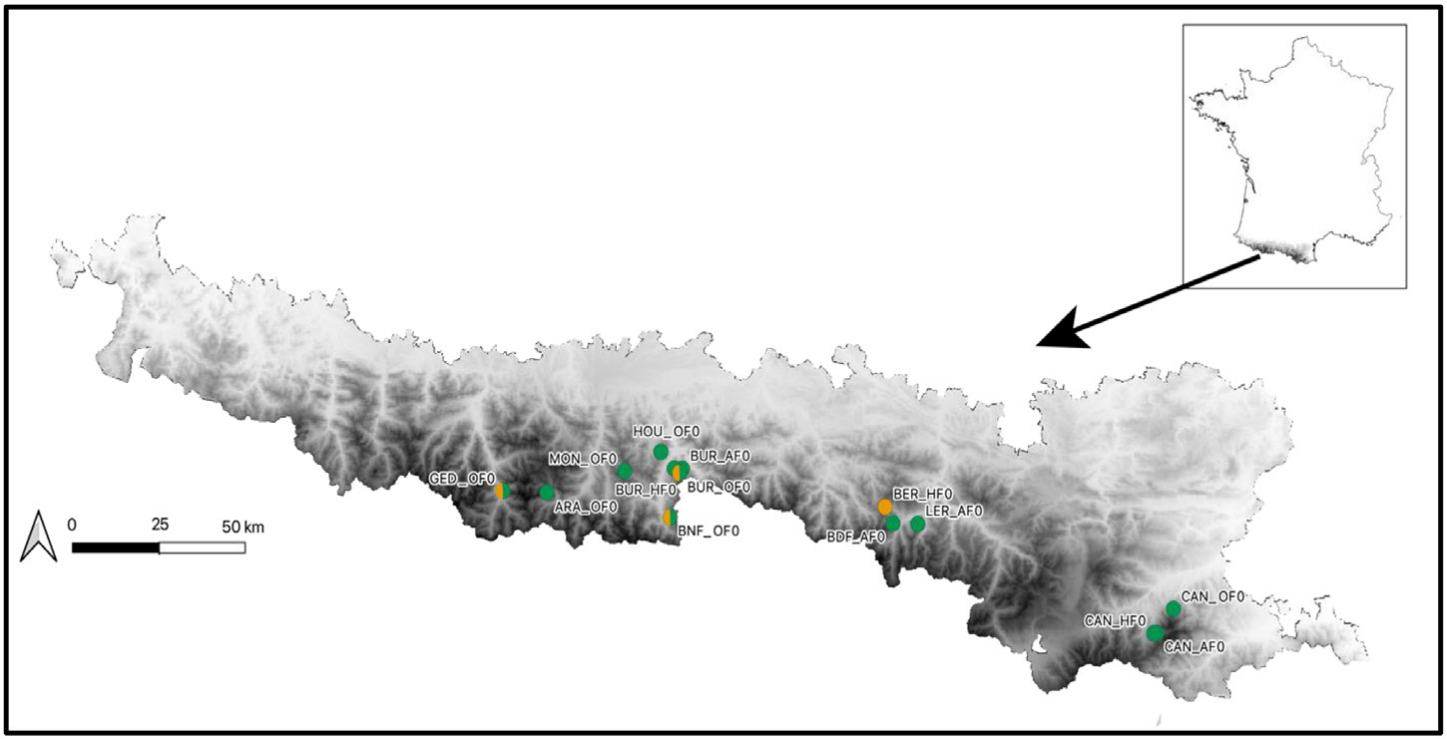
The map illustrates the distribution of all forest stands studied in the French Pyrenees. The dark green dots represent fir populations, while the orange dots represent beech populations. In stands where both species were sampled, the dots are placed side by side.

**Fig. 2:**
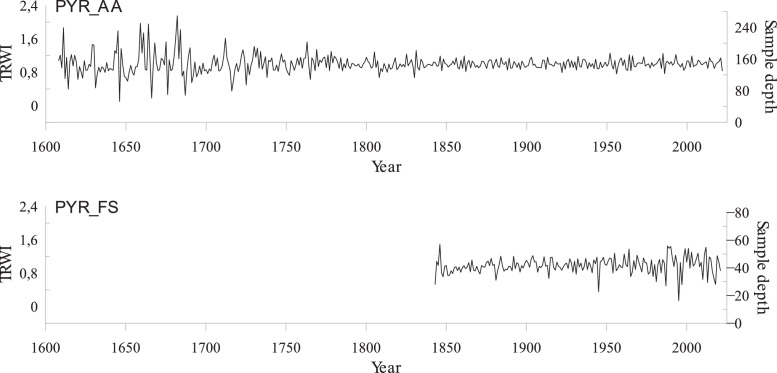
Detrended reference chronologies of fir (top) and beech (bottom) in the Pyrenees. An age-dependent spline was fitted to the raw individual tree-ring series, and then the individual tree-ring indices (TRWi) were calculated by dividing raw measured values by the fitted ones. Pre-whitened TRWi were averaged to build reference chronologies. We also plot the sample depth of the two chronologies.

**Fig. 3:**
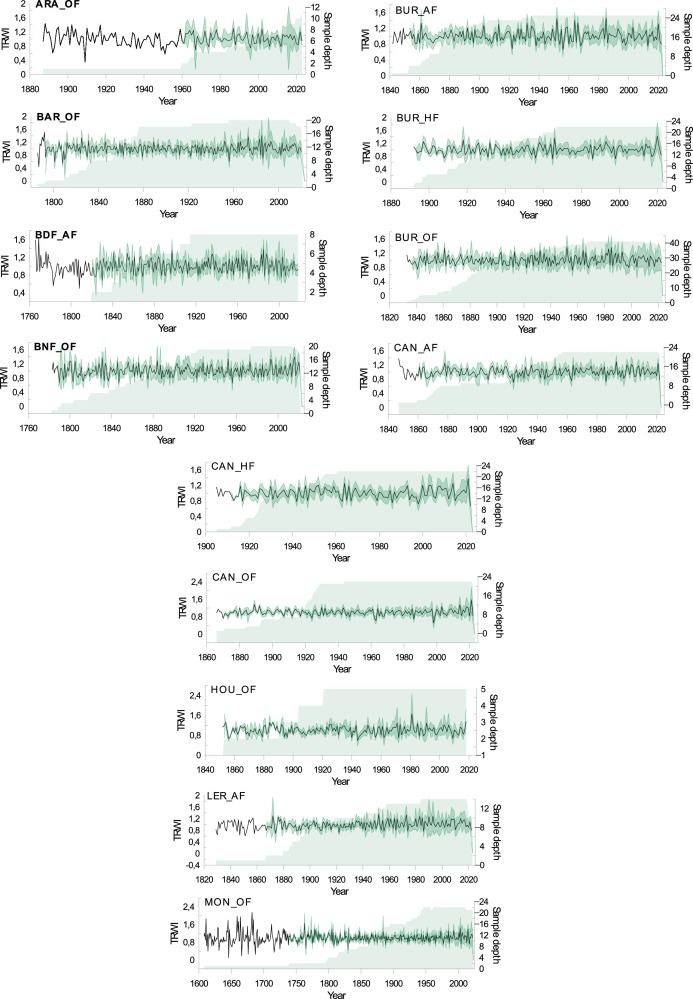
Detrended master stand chronologies of **fir.** An age-dependent spline was fitted to the raw individual tree-ring series, and then the individual tree-ring indices (TRWi) were calculated by dividing raw measured values by the fitted ones. Pre-whitened TRWi were averaged to build reference chronologies. We also plot the sample depth of the two chronologies. Please pay close attention to the scale of the abscissa (years), which varies according to the chronology.

**Fig. 4:**
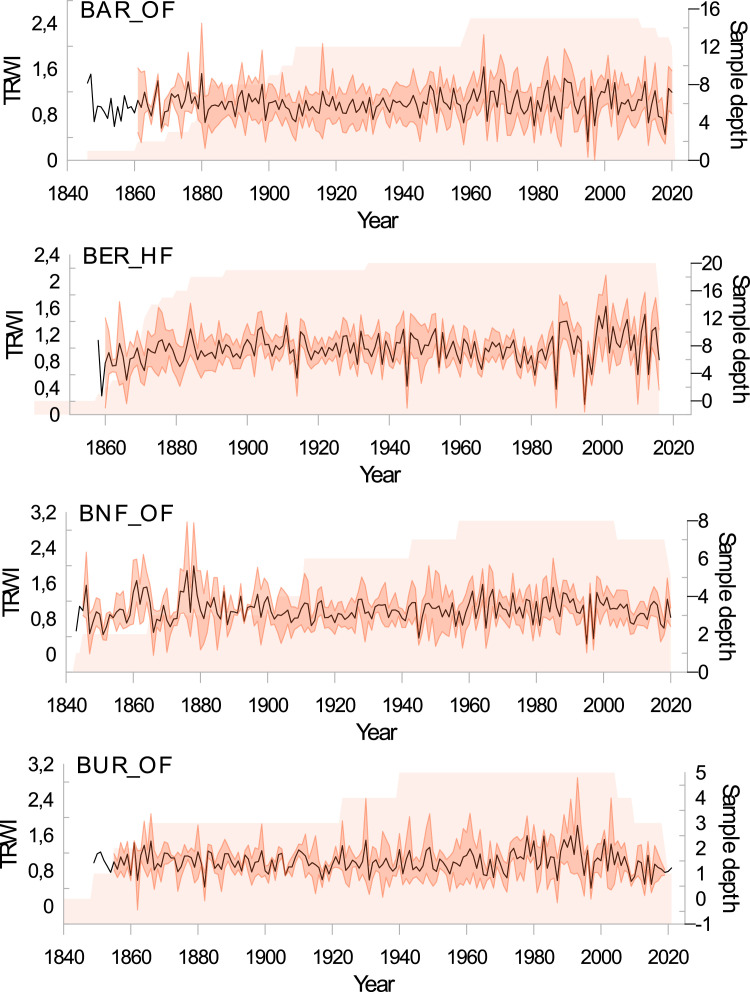
Detrended master stand chronologies of **beech**. An age-dependent spline was fitted to the raw individual tree-ring series, and then the individual tree-ring indices (TRWi) were calculated by dividing raw measured values by the fitted ones. Pre-whitened TRWi were averaged to build reference chronologies. We also plot the sample depth of the two chronologies. Please pay close attention to the scale of the abscissa (years), which varies according to the chronology.
• (4) A folder “R script” contain a R script for reading and performing several operations on the master stand and reference chronologies in the open-source software R using the 'dplR' and 'detrendR' packages.
• (5) A Read me in text format (.txt) provides explanations to easily navigate and use the data provide in this repertory.

The reference chronologies for fir (Top panel) and beech (bottom panel) are plotted in Fig. 2.

Table 2 gives standard statistical data for dendrochronology. These values are calculated either on raw chronologies or on detrended chronologies (see section: Experimental design, materials and methods).

Finally, the Table 3 (not included in the data repertory) provides all information about the data included in the files associated with this data paper (DOI: 10.48579/PRO/JQQRRK).

**Table 3:** The following table illustrates the tree structure of the data folder associated with this data paper.

*Main folder*	*Folders*	*Subfolders 1*	*Subfolders 2*	*Files*	*Contents*
*Data*	*-*	*-*	*-*	*Read_me.docx*	Explanations to easily navigate and use the data provided in this repertory
	*Figures (.pdf)*			Fig. 1	A map of the study area
				Fig. 2	A figure with detrended reference chronologies for fir and beech plotted with the standard deviation and the sample depth
				Fig. 3	A figure with all detrended master stand chronologies for fir plotted with the standard deviation and the sample depth. Please pay close attention to the scale of the abscissa (years), which varies according to the chronology.
				Fig. 4	The same information as in Fig. 3, but with beech chronologies this time
	*Rscript (.R)*	*-*	*-*	*Read_datafiles*	A typical R script that provides a step-by-step explanation of the code required to open individual series of all stands in R, calculate statistics and detrend chronologies with the aim to remove noise signals. In this way, we hope to convince people who are unaccustomed to working with dendrochronological data in R
	*Tables (.tab)*	*-*	*-*	Table 1	The file entitled 'Description of the chronologies' provides comprehensive information regarding the forest stands from which the samples were obtained for the construction of all master stand chronologies. The altitude, latitude and longitude are derived from the average location of the sampled trees. The degree of human pressure is divided into three factors: high forest (HF), ancient forest (AF) and old growth (OF). The table also contains the number of individuals cored as well as the number of individuals retained to construct the chronology.
				Table 2	The file entitled 'Chronology statistics' provides the primary statistical data pertaining to raw and indexed chronologies (the detrending method applied is the'AgeDepSpline' from the DplR package in the free R software). Regarding raw data, we calculate mean, standard deviation and first order autocorrelation of the master stand and reference chronologies (``mean'',``stdev'', “Ar1”).. Detrending were applied to calculate the rbar total, which depict the mean of all the correlations between different cores, and the expressed populations signals which allow to assess the common signal recorded in a chronology. Additional statistical data, as provided in R's “dplR” package, can be generated using the R script associated with the data in the directory of storage.
	*Individual series and chronologies*	*Individual series for stand and Pyrenees*	*Format CSV*	*Abies alba*	A folder containing all well-cross-dated individual fir series in raw data used to build master stand and reference chronologies. The folder therefore includes 14 files in .csv format.
				*Fagus sylvatica*	A folder containing all well-cross-dated individual beech series in raw data used to build master stand and reference chronologies. The folder therefore includes 5 files in .csv format.
			*Format FH*	*Abies alba*	A folder containing all well-cross-dated individual fir series in raw data used to build master stand and reference chronologies. The folder therefore includes 14 files in .fh format.
				*Fagus sylvatica*	A folder containing all well-cross-dated individual beech series in raw data used to build master stand and reference chronologies. The folder therefore include 5 files in .fh format.
		*Chronologies*	*-*	*Master stand and reference chronologies_AA*	Fir master stands and Pyrenees reference chronologies as calculated with the Rcode also provided in Tucson (.rwl) or tabular format (.csv)
				*Master stand and reference chronologies_FS*	Beech master stands and Pyrenees reference chronologies as calculated with the Rcode also provided in Tucson (.rwl) or tabular format (.csv)

## 4 Experimental Design, Materials and Methods

The studied stands are located in the central northern Pyrenees of France, in the montane and subalpine belts, in the departments of *Ariège, Haute-Garonne,Hautes-Pyrénées and Pyrénées orientales* (Fig. 1). They are either beech- or fir-dominated forests, or mixed fir-beech forests, in which other species coexist more or less discreetly, such as mountain ash (*Sorbus aucuparia* L.), sycamore maple (*Acer pseudoplatanus* L.), and birch (*Betula pendula* Roth. and *Betula pubescens* Ehrh). Altitude of sampled stands ranges from 1,400 to 1,800 m a.s.l. The forest stands are distributed along a drought gradient running from west (Gèdre for the two species) to east (Canigou for fir, and Bernadouze for beech). The stand aspect is mainly northern.

In Burat and Canigou, three distinct stands were identified according to the levels of recent and past silvicultural pressure. These included forests managed as regular high standard forests (HF), ancient forests where harvesting ceased at least 50 years ago (AF), and old-growth forests where no further harvesting has taken place for over 100 years (OF). It should be noted that some of the formers are also subject to strict reserve status (Table 1).

### 4.1 Sampling strategies

The sampling strategy varied according to the project context in which the chronologies were acquired. In fact, these data are the result of several projects with different objectives. In Bernadouze, the FODYNA projects (2013-2018) supported by the “*Observatoire Homme-Milieu*” (OHM, https://www.driihm.fr/la-recherche/projets-ohm-et-interohm?view=projet&id=330), funded by the Labex “*Dispositif de Recherche interdisciplinaire sur les Interactions Hommes-Milieux*” (DRIIHM), and Léonel Fouédjeu's PhD thesis aim, inter alia [6], to refine the precise chronology of the former charcoal manufacturing activity mainly related to mining and smelting activities, which has been studied by archaeological and anthracological approaches [4]. A charcoal chronology was built, but due to the lack of beech reference chronology in Pyrenees, it was necessary to create one new reference chronology for beech growing in the local area. The beech trees were sampled during the forest logging managed by the National Forests Office (ONF) in the autumn 2016. Trees to sample were selected according to their estimated age (i.e selection of oldest/largest trees) and their type (standard beech trees, we excluded coppice trees). Sixty-four cross-sections were extracted by lumberjacks directly from the stumps of freshly felled trees [4]. The fir trees used to build the master chronologies from the state forest of Lercoul and from the protected forest of “Bois du Far” were randomly selected among largest trees to estimate overall stand tree ages along an altitudinal gradient as part of the TRANSYLVE 1 to 2 projects (2019-2021), which represents a continuation of the FODYNA 1 to 6 projects [7,8], (https://www.driihm.fr/la-recherche/projets-ohm-et-interohm?view=projet&id=2165, also supported by the OHM Haut-Vicdessos and funded by the Labex DRIIHM. The dendrochronological approach carried out in the framework of the BENDYS project (ANR-19-CE03-0010; https://anr-bendys.cnrs.fr) aim to well-characterize the sub-recent dynamic of old-growth fir-beech forest and to assess the resilience capacity of both species to current global changes [1]. In the three OGF stands, Burat, Bois Neuf and Barrada, and in the wooden pasture of Hourmigué, at least fifteen individual fir and beech trees (no beech trees were sampled in Hourmigué) were designated according to their size and location within the stands resulting in four master stand chronologies of fir and 3 for beech. The OcciGen project (ink:href="https://occigen.hub.inrae.fr) funded by the Occitanie region aims to better characterize the ability of the main forest species of economic interest to adapt to global change according to different modalities and different degrees of forest management. This project led by INRAE (Avignon) is a continuation of the BENDYS project, which is why one of the sites studied in OcciGen is the same as in BENDYS. The combination of the two projects has made it possible to acquire many more fir core samples in the old-growth forest of Burat. In the framework of the OcciGen project, we also built additional five new chronologies for fir in Burat and in the Canigou forest. Finally, in the frame of the BOSCA project (https://sciencesdupasse.univ-toulouse.fr/projet-bosca/, also funded by the Occitanie Region, one fir-dominated OGF and one fir-dominated ancient forest stands were selected: the Montious nature reserve and the Aragnouet public forest. The aim of this project was to find fir trees old enough to allow the dating of chronologies obtained from buildings along the Garonne as far as Montauban and test for dendroprovenancing.

With the exception of the beech trees sampled in Bernadouze by a lumberjack and benefiting from an ONF-organized forestry cut, the other trees were cored at breast height using an increment borer (Haglof borer 400 mm to 1000 mm). To maximize the chances of reaching the pith, one or two cores were taken from each tree. According to the project, we cored 15 to 64 trees of one or of the two species in all stands. In total, we sampled 309 fir trees in 13 stands and 115 beech trees in 4 stands.

### 4.2 Laboratory treatment

Cross-sections and cores were first air dried. The cores were glued to wooden sticks, sometimes with the aid of a binocular loupe, to reveal the transversal section at the top. All samples were sanded with a belt sander of various grits (P80 to 600) to magnify the tree ring borders then observed under a binocular loupe for accurate measurement. Prior to measurement, the samples were first observed and the rings counted from the bark to the pith (if present) of the samples. Pointer-years rings were noted to compare the samples with each other and to highlight any early crossdating errors. For most of the dataset, tree ring-widths were measured using the incremental measuring table LINTAB-6 with 1/100 mm accuracy connected with the TSAP-Win software [9]. The cores from Burat, Canigou, Montious and Aragnouet were scanned at a high resolution (2,400 dpi true resolution) with an Epson Expression 12,000 XL scanner. Tree ring-width measurements were then performed on scanned images using the software CooRecorder/CDendro [2] (Version 9.6, Cybis Elektronik & Data AB, Sweden) with a precision of 0.01 mm ^2^.

The last ring of all chronologies is the last measurable one, i.e. the one including a complete initial and final wood. If the individual chronology shows a last ring that differs from the year in which the stand was harvested, this means that the last ring or rings were no longer present on the measured core, or that the correlation was very poor. Individual chronologies were then automatically normalized in the C-DENDRO software using the P2yrs methods [2]. The cross-dating accuracy was derived from the Student t-test (t) by considering a threshold value of 3.5 [10,11]. To assess the cross-dating quality, we used the long-term fir chronology built with archaeological materials for the Gedre Valley [5] and the two beech reference chronologies from Baish Aran (in the central Spanish Pyrenees) and Iraty (in the eastern French Pyrenees) [12,13]. We also used the data from the REMOTE Primary Forest database to assess the cross-dating quality for the stands Burat, Bois-neuf and Barrada (https://www.remoteforests.org). Visual cross-dating was repeated several times while utilizing the Math graph tool in CDendro to improve statistics. This cross-dating step is crucial to ensure the robustness of the chronology and, if necessary, to correct errors in individual chronologies (missing rings, double rings, etc.). The number of individuals retained for the construction of chronologies (i.e. individual tree-ring series with a t >3,5) is given in the “Chronology statistics.csv” file in the associated files (Table 3). We applied the same step-by-step process to build all master stand chronologies, we averaged well-cross-dated individual raw series using a bi-weighted robust mean to build master stand and reference chronologies. Table 3 provides the mean and standard deviation of all master stand and reference chronologies, and the first order autocorrelation, which assess the influence of the previous year growth upon the current year growth [14].

We finally detrended the chronologies to remove noise signals and calculate additional statistics, namely the total mean inter-series correlation (rbar_tot_) and the expressed population signal (EPS), to explore the strength of the common signal shared by the population. To calculate all individual indexed series (TRWi), we fitted an age-dependent spline (“*AgeDepSpline*”) using the ‘detrend’ function in the *dplR* [15] and *detrendR* [16] packages in the R software, to the individual raw series, and calculate a year-to-year ratio between raw and indexed series. We then applied an autoregressive filter (pre-whitened) to remove the high first order autocorrelation observed in all chronologies (AR1 > 0). TRWI series were then averaged using a bi-weighted robust mean to build indexed master stand chronologies and Pyrenees reference chronologies (Fig. 1).

## Limitations

The master stand chronologies of Hourmigués (Hou) for fir and Bois Neuf (BNF) for beech are based on a limited number of individuals, which renders them somewhat fragile and prone to interannual variability. Nevertheless, these chronologies demonstrate a high degree of correlation with other Pyrenean chronologies, and thus we assume them to be valid. New samples can be taken in the future to strengthen them. The Aragnouet and Pyrenees fir chronologies, which date back to 1605, are based on just one individual and should therefore be treated with caution. Nevertheless, we decided not to cut this individual series, given its strong correlation with the fir chronology built using archaeological materials [5]. The rbar (mean interseries correlation) values range from 0.204 (reference chronologies) to 0.393 (master chronology of the ancient forest of Burat) for fir, and between 0.215 (master chronology of the OF of Burat) and 0.394 (master chronology of the MF of Bernadouze) for beech. For fir, this range of rbar values is commonly observed and likely reflects significant common variations in response to climatic factors [15]. In contrast, for beech, the values are relatively low compared to other studies. The EPS values derived from the fir tree chronologies are predominantly above the threshold of 0.85, indicating a robust common signal, with the exceptions of the Bois du Far and Hourmigué chronologies [16]. This means that most of them are suitable for dendroclimatological purposes. With regard to the beech chronologies, only the master chronology of Bois Neuf and the reference chronology for the Pyrenees exceed this threshold. The reduced EPS and rbar values for beech may be attributed to its co-dominant status in most plots, with the exception of Bernadouze, suggesting that growth variations are likely primarily influenced by within-site competition. For this reason, we advise that beech master chronologies, but not the reference, be used with caution for all analyses relating to dendroclimatology.

## Ethics Statement

The authors certify that they have read and complied with the ethical requirements for publication in Data in Brief and confirm that the current work does not involve human subjects, animal experiments or data collected on social media platforms.

## CRediT Author Statement

**Melanie Saulnier:** Conceptualization, Methodology, Software, Validation, Formal analysis, Data curation, Writing- Original draft preparation, Writing- Reviewing and Editing, Supervision. **Vincent Labbas:** Methodology, Software, Validation. **Roberta D’andrea:** Methodology, Software, Validation, Formal analysis, Data curation, Writing- Reviewing and Editing. **Sylvain Burri:** Conceptualization, Methodology, Writing- Reviewing and Editing, Funding acquisition, Project administration. **Laurent Larrieu:** Conceptualization, Methodology Writing- Reviewing and Editing. Sylvain Morvan: Formal analysis, Data curation. **Nicolas Martin:** Conceptualization, Methodology, Validation, Formal analysis, Writing- Reviewing and Editing. **Caroline Scotti-Saintagne:** Conceptualization, Methodology, Funding acquisition, Project administration. **Frédéric Jean:** Conceptualization, Methodology, Funding acquisition, Project administration. **Poirier Nicolas:** Funding acquisition, Project administration. **Vanessa Py-Saragaglia:** Conceptualization, Methodology, Funding acquisition, Project administration, Writing- Reviewing and Editing.

## Data Availability

DataverseOriginal dataset of master stand and reference chronologies for living Silver fir (Abies alba Mill.) and European beech (Fagus sylvatica L.) in the French Pyrenees (France) (Original data).
